# FEASIBILITY OF A COMMUNITY-LEVEL PHYSICAL ACTIVITY STRATEGY FOR OLDER ADULTS WITH MOTORIC COGNITIVE RISK SYNDROME

**DOI:** 10.1093/geroni/igad104.1264

**Published:** 2023-12-21

**Authors:** Kieran Reid

**Affiliations:** Brigham and Women’s Hospital/Harvard Medical School, Boston, Massachusetts, United States

## Abstract

The motoric cognitive risk syndrome (MCR) is a pre-dementia syndrome characterized by slow walking speeds and subjective memory complaints. To date, the feasibility of a community-based physical activity (PA) program for improving outcomes in MCR has not been examined. To address this knowledge gap, we conducted a translational randomized controlled trial (RCT) of 24-weeks of PA or a healthy aging education (HE) control intervention delivered within the existing infrastructure of an urban senior center in Greater Boston. A senior center employee was trained to administer the multimodal group-based PA program that included moderate-intensity aerobic walking, strength, flexibility and balance training. A total of 79 older adults attended the senior center for a screening visit of whom 25 met the MCR criteria and were randomized to PA or HE (mean age: 74.4 ± 7 yrs; BMI: 32.4 ± 7 kg/m2; 85% female; 3MSE score: 92.4 ± 7; gait speed: 0.52 ± 0.1 m/sec; SPPB score 4.8 ± 1.9). Due to COVID-19 the study was terminated early, however participants successfully completed baseline and follow-up study assessments that included objective tests of physical function and an iPad-based cognitive testing battery. Participants could appropriately adhere to the study interventions (overall attendance rate: PA: 69.3% vs. HE:70.1% at study termination). No study-related adverse events occurred. Trends for improved executive cognitive performance were exhibited in PA compared to HE. Our study provides important preliminary information to aid the design of larger-scale RCTs to preserve the independence of vulnerable older adults with MCR in community-based settings.

